# Interpretable machine learning to identify important predictors of birth weight: A prospective cohort study

**DOI:** 10.3389/fped.2022.899954

**Published:** 2022-11-11

**Authors:** Zheng Liu, Na Han, Tao Su, Yuelong Ji, Heling Bao, Shuang Zhou, Shusheng Luo, Hui Wang, Jue Liu, Hai-Jun Wang

**Affiliations:** ^1^ Department of Maternal and Child Health, School of Public Health, Peking University, National Health Commission Key Laboratory of Reproductive Health, Beijing, China; ^2^ Tongzhou Maternal and Child Health Care Hospital of Beijing, Beijing, China; ^3^ Department of Epidemiology and Biostatistics, School of Public Health, Peking University, Beijing, China

**Keywords:** birth weight, predictor, interpretable machine learning, cohort, pregnancy

## Abstract

**Background:**

Predicting birth weight and identifying its risk factors are clinically important. This study aims to use interpretable machine learning to predict birth weight and identity important predictors.

**Methods:**

This prospective cohort study was conducted in Tongzhou Maternal and Child Health Care Hospital of Beijing, China, recruiting pregnant women between June 2018 and February 2019. We used 24 features to predict infant birth weight, including gestational age, mother's age, parity, history of macrosomia delivery, pre-pregnancy body mass index (BMI), height, father's BMI, lifestyle (diet, physical activity, smoking), and biomarker (fasting glucose and lipids) features. Study outcome was birth weight of infant. We used 8 supervised learning models including 4 individual [linear regression, ridge regression, lasso regression, support vector machines regression (SVR)], and 4 ensemble estimators (random forest, AdaBoost, gradient boosted trees, and voting ensemble for regression) to predict birth weight. Model accuracy was measured by root mean squared error (RMSE) of 10-fold cross validation on the training set and RMSE of prediction on the test set. We used permutation importance algorithm to understand the prediction from the models and what affected them.

**Result:**

This study included 4,754 mother-child dyads. RMSEs were lower in voting ensemble for regression, linear regression, and SVR than random forest, AdaBoost, and gradient boosted tree. The 5 most important predictors for infant birth weight were gestational age, fetal sex, preterm birth, mother's height, and pre-pregnancy BMI. After adding ultrasound-measured indicators of fetal growth into predictors, mother's height and pre-pregnancy BMI remained the most important predictors in predicting the outcome.

**Conclusion:**

Mother's height and pre-pregnancy BMI were identified as important predictors for infant birth weight. Interpretable machine learning is a promising tool in the prediction of birth weight.

## Introduction

Prediction of birth weight is clinically important. In the short term, low birth weight increases the risk of stillbirth, preterm birth, intrapartum-related events, and neonatal death (1); in the long term, individuals with low birth weight have a higher risk of developing cardiovascular disease and adult depression (2), based on the Developmental Origins of Health and Disease theory (3). Macrosomia, lying at the other end of birth weight, is not only associated with an immediate risk of shoulder dystocia, cesarean section, and neonatal hypoglycemia, but also heightens the risk of obesity and diabetes in the period of childhood and adolescence (4). If important predictors of birth weight were identified, targeted interventions can be timely implemented among high-risk subpopulations.

Genetic, environmental, and gestational factors can affect the size of birth weight. Complex non-linear relationship or interactions might exist in high-dimensional data, making it difficult for conventional linear models to accurately predict birth weight. Machine learning methods, widely used in biomedical research (5), might be a promising tool in predicting the birth weight. A recent systematic review recommended researchers to use both linear regression and other machine learning models to predict pregnancy outcomes (6).

Machine learning is analogous to “a black box” due to its unintuitive interpretability in early years. Recent progress in methodology has made machine learning both predictable and interpretable (7, 8). This study aims to predict birth weight and examine its important predictors by using interpretable machine learning methods.

## Materials and methods

### Study design and population

The Peking University Birth Cohort in Tongzhou (PKUBC-T) was a prospective cohort study conducted in Tongzhou Maternal and Child Health Hospital of Beijing, China. This cohort was prospectively registered in ClinicalTrials.gov (https://clinicaltrials.gov/, NCT03814395), and aimed to study the health effects of pre-pregnancy and prenatal exposures on mother-child dyads. Pregnant women were recruited between June 2018 and February 2019 at baseline. Eligibility criteria were: (1) aged 18–45 years; (2) <14 weeks of gestation; (3) living in Tongzhou District during the past half year and not planning to move out of Tongzhou District after delivery; (4) planning to receive prenatal care and give birth in Tongzhou Maternal and Child Health Hospital. A total of 5,426 eligible pregnancy women were recruited into the cohort. Ethical approval of the study was granted by the Peking University Institution Review Board (IRB00001052-18003). Written informed consent was obtained from all participants.

For the present study, we included pregnant women with singleton live births (*n* = 4,798), excluded those with diabetes or hypertension prior to pregnancy (*n* = 44), and finally included 4,754 mother-child dyads.

### Predictors

We selected predictors (features) based on literature review of studies on this topic (6, 9). We finally included 24 predictors with available data and satisfactory completeness (missing <20%) in our cohort. Information of 11 predictors were collected from face-to-face questionnaire investigation at the first prenatal visit, including age (year), parity (0, ≥1), history of macrosomia delivery (yes, no), pre-pregnancy body mass index (BMI = body weight/square of height; kg/m^2^), height (cm), father's BMI (kg/m^2^), family income last year (yuan), smoking in the last 3 months (yes, no), dietary energy intake per day (kcal; recording dietary intake for 2 non-consecutive days and calculating the average energy intake), alcohol consumption (yes, no), number of days performing moderate to vigorous physical activity (MVPA) per week. Information of 6 predictors were collected from the hospital information system with vigorous quality control: gestational diabetes (yes, no), gestational hypertension (yes, no), eclampsia/preeclampsia (yes, no), preterm birth (yes, no), gestational age, and fetal sex (male, female). Information of the other 7 predictors were collected from blood samples, including fasting plasma glucose concentrations (mmol/L) and 25(OH)D_3_ (ng/mL) measured between 24 and 28 gestational weeks, fasting concentrations of triglyceride (mmol/L), cholesterol (mmol/L), hemoglobin (g/L), thyroid-stimulating hormone (TSH; mIU/l), and free thyroxine (FT4; pmol/L) measured in the first trimester (<14 gestational weeks). In the sensitivity analyses, we added ultrasound-measured indicators of fetal growth (abdominal circumference, head circumference, femur length, and biparietal diameter) collected before 14 gestational weeks as the predictors.

### Outcome

Study outcome was infant birth weight. We obtained it from the hospital information system in Tongzhou Maternal and Child Health Care Hospital of Beijing.

### Machine learning

We used a tool of scikit-learn 0.24 (10) to conduct machine learning in Python 3.8.5.

**Figure 1:**
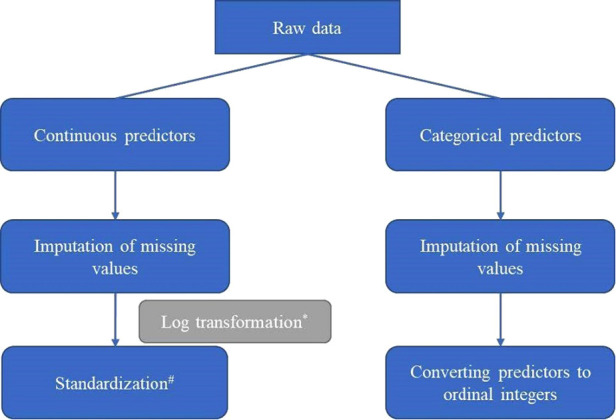
Pipeline of preprocessing data (*Log transformation was conducted for predictors with skew distribution; ^#^Standardization: transforming data into normal transformation with zero mean and unit variance.).

We first preprocessed data (e.g., imputation, transformation, standardization; Figure 1) and randomly divided it into a training set and a test set according to 4 : 1. Then we used the training set to train 8 supervised learning models including individual and ensemble estimators (11). Individual estimators included linear regression, ridge regression, lasso regression and support vector machines for regression (SVR). Ensemble estimators, aggregating a group of several base estimators by bagging or boosting methods, aimed to improve generalizability of robustness over a single estimator. We used random forests (bagging method), AdaBoost (boosting method), and gradient boosted trees (boosting method) that were trained based on a group of decision trees (base estimators). Except for linear regression, we used a combination of grid search and cross validation to select model hyperparameters when training other machine learning models. For each model, we tried more than 500 hyperparameter combinations to select the best hyperparameter combination and trained the final regressors. We also trained a voting ensemble for regression by combining the 7 final regressors (linear regression, ridge regression, lasso regression, SVR, random forest, AdaBoost, gradient boosted trees) and obtaining the average predicted values.

Root mean squared error (RMSE) was used to measure the accuracy of the model. Lower value of RMSE indicated better performance of the model.


$$\mathrm{RMSE}\;\left(y,\hat{y}\right)=\;2\sqrt{\frac{1}{n_{\mathrm{samples}}}\overset{n_{\mathrm{samples}}-1}{\underset{i=0}{\sum }}{\left(y_{i}-\hat{y_{i}}\right)}^{2}}$$

($\hat{y_{i}}$ is the predicted value of the *i*-th sample, and $y_{i}$ is the corresponding true value).

We evaluated model performance on both training and test datasets. On the training set, model performance was evaluated by using the 10-fold cross validation. First, the training dataset was split into 10 smaller sets. Second, a model was trained using 9 of the folds as training data. Third, the resulting model was validated on the remaining part of the data and RMSE was calculated. Last, the average of the 10 RMSE values was calculated to evaluate model performance. On the test set, we used the final regressors obtained from the training set to predict the birth weight and calculate the RMSE.

To increase the interpretability of machine learning models, we further evaluated how much the model depended on the feature (i.e., feature importance) by the permutation-based method in two steps. First, a baseline score (RMSE) was calculated for the final model. Second, a feature is permutated (randomly shuffled) from the dataset to break the association of the feature with the target, and the model evaluation score was calculated again to obtain the permutation score. The second step was repeated for 50 times. The permutation-based feature importance was calculated as the mean difference between the baseline score and the permutation score; that is, a larger difference indicates the higher importance of the feature in the model. We also used partial dependence plots to visualize the direction and size of associations (i.e., positive or negative) between the outcome and the inputted features, marginalizing over the values of all other input features. The interpretability methods used above were all carried out on the test datasets.

## Results

The average infant birth weight was 3362.0 g [standard deviation (SD): 471.8]. The description of study sample is shown in Table 1.

**Table 1:** Description of 4,754 mother-child dyads.

Variables	Description
**Continuous variables** ^a^	
Age, year	29.3 ± 3.8
Height, cm	160.1 ± 5.1
Pre-pregnancy BMI, kg/m^2^	22.5 ± 3.4
Father's BMI, kg/m^2^	22.5 ± 5.5
Family income, 10,000 yuan/year	15 (10–20)
Energy intake, kcal/day	1,262.4 (967.5–1,655.2)
Glucose, mmol/l	4.8 ± 0.4
Triglyceride, mmol/l	1.1 (0.8–1.4)
Cholesterol, mmol/l	4.0 ± 0.7
Infant birth weight, g	3,362.0 ± 471.8
Infant birth weight *Z*-score	0.3 ± 1.0
**Categorical variables, *n* (%)**	
Energy intake > 1,800 kcal/day	840 (17.7%)
Weekly MVPA	944 (18.1%)
Primipara	2,892 (60.8%)
History of macrosomia delivery	125 (2.6%)
Smoking	467 (9.9%)
Alcohol consumption	172 (3.6%)
Gestational diabetes	1,522 (32.0%)
Gestational hypertension	141 (3.0%)
Eclampsia/preeclampsia	209 (4.4%)
Preterm birth	229 (4.8%)
Female fetuses	2,307 (48.5%)

BMI, body mass index; MVPA, moderate to vigorous physical activity.

^a^ Described as mean ± standard deviation, for variables with normal distribution; described as median (inter-quartile), for variables with skew distribution.

**Figure 2:**
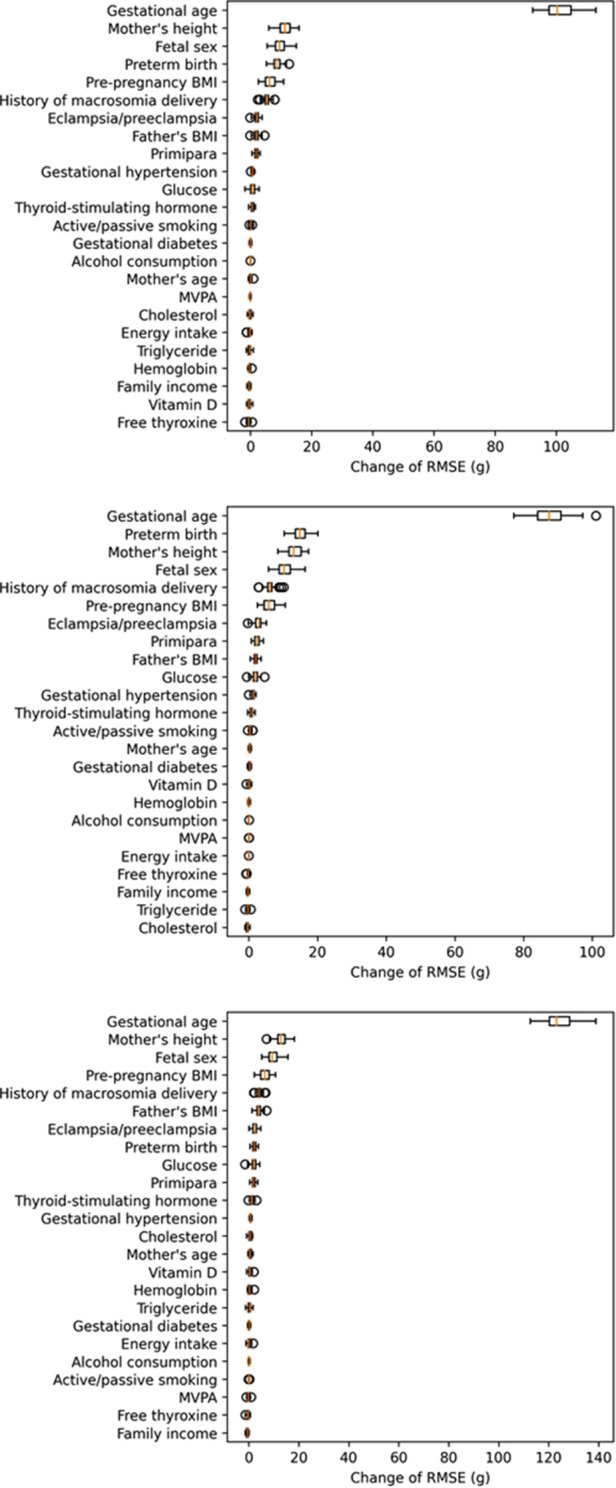
Permutation importance of predictors in the voting ensemble for regression (top figure), linear regression (middle figure), and SVR (bottom figure) models (calculation of permutation importance score was repeated for 50 times, and the box-plot for each predictor showed the distribution of 50 permutation scores).

As shown in Table 2, among the 8 machine learning models, the voting ensemble regression had the highest accuracy, followed by linear regression (simple linear regression, ridge regression and lasso regression), support vector machine regression, random forest, AdaBoost, and gradient boosted trees. The 5 most important predictors were gestational age, fetal sex, preterm birth, mother's height, and pre-pregnancy BMI. Results of permutation importance of predictors based on voting ensemble for regression, support vector machine regression, and linear regression models are shown in Figure 2; results of permutation importance based on other models are shown in Supplementary Figure S1. The 5 most important predictors for birth weight remained similar in the sensitivity analyses of excluding preterm-birth and/or small-for-gestational-age infants (*n* = 275), except that the predictor of preterm birth was replaced by the history of macrosomia (Supplementary Figure S2).

**Table 2:** Root mean squared error and the 5 most important predictors for the prediction of infant birth weight using 8 machine learning models.

	LR	Ridge	Lasso	RF	AdaBoost	GBT	SVR	VER
**RMSE of cross-validation** ^a^	372.49 ± 13.64	372.47 ± 13.76	372.40 ± 13.69	377.05 ± 13.78	379.05 ± 14.96	383.50 ± 12.53	372.71 ± 14.43	369.99 ± 13.46
**RMSE** ^b^	377.11	377.16	377.22	381.69	385.33	383.14	373.83	374.89
**Predictors**								
** **Gestational age	√	√	√	√	√	√	√	√
** **Mother's height	√	√	√	√	√	√	√	√
** **Fetal sex	√	√	√	√	√	√	√	√
** **Pre-pregnancy BMI		√	√	√	√	√	√	√
** **Preterm birth	√	√	√	√			√	√
** **History of macrosomia delivery	√				√		√	
** **Father's BMI						√		
** **Eclampsia/preeclampsia	√						√	

BMI, body mass index; GBT, gradient boosted trees; LR, linear regression; RMSE, root mean squared error; RF, random forest; SVR, support vector machines regression; VER, voting ensemble for regression.

^a^ Obtained by cross-validation in the training set (*n* = 3,803) during model training (mean ± standard deviation).

^b^ RMSE of the final model when making predictions on the test set (*n* = 951).

**Figure 3:**
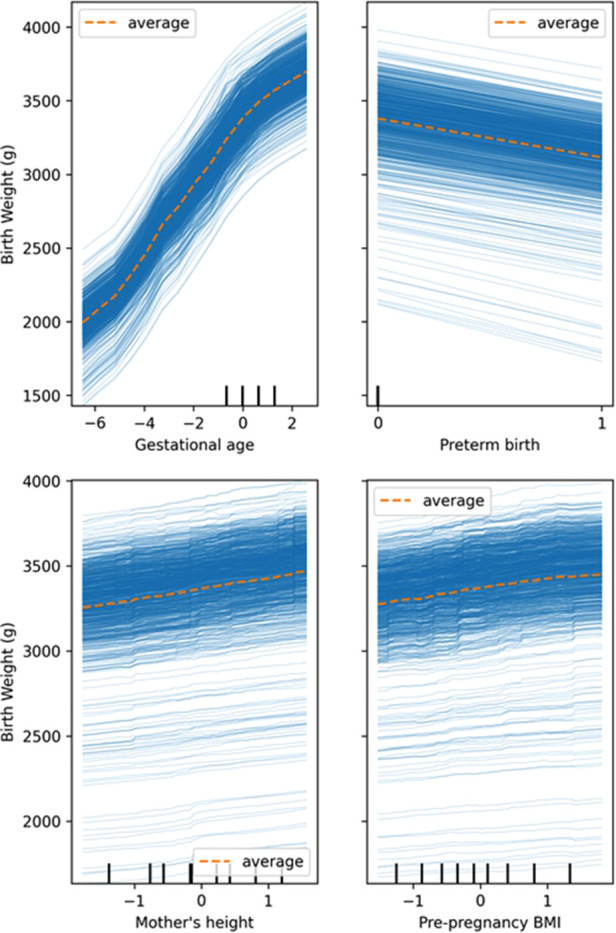
Partial dependence plots of gestational age, preterm birth, mother's height, and pre-pregnancy BMI, in the prediction of birth weight in the voting ensemble for regression model (gestational age, mother's height, and pre-pregnancy BMI were standardized; other models in the Supplementary File).

As shown in partial dependence plots (Figure 3, Supplementary Figures S3–S8), mother's height and pre-pregnancy BMI were positively associated with birth weight. And logically, birth weight is positively correlated with gestational age, with preterm babies having lower birth weights. Pattern of associations between these predictors and birth weight was approximately linear in the nonlinear models of voting ensemble for regression (Figure 3), random forests (Supplementary Figure S6), AdaBoost trees (Supplementary Figure S7), and gradient boosted trees (Supplementary Figure S8).

Accuracy of all 8 models was improved after adding ultrasound-measured indicators of fetal growth into predictors (Table 3). The 5 most important predictors were gestational age, preterm birth, ultrasound-measured fetal abdominal circumference, mother's height, and pre-pregnancy BMI (Table 3).

**Table 3:** Root mean squared error and the 5 most important predictors for the prediction of infant birth weight using 8 machine learning methods after considering ultrasound-measured fetal growth.

	LR	Ridge	Lasso	RF	AdaBoost	GBT	SVR	VER
**RMSE of cross-validation** ^a^	353.64 ± 13.51	353.62 ± 13.62	353.51 ± 13.63	361.79 ± 15.71	361.20 ± 14.08	367.24 ± 12.86	353.53 ± 15.01	352.05 ± 14.85
**RMSE** **^b^**	351.90	351.95	352.06	363.16	364.63	369.04	350.86	352.49
**Predictors**								
Gestational age	√	√	√	√	√	√	√	√
** **Abdominal circumference	√	√	√	√	√	√	√	√
Pre-pregnancy BMI	√	√	√		√		√	
Mother's height	√	√	√	√	√	√	√	√
Preterm birth	√	√	√	√				√
Head circumference				√	√	√	√	√
History of macrosomia delivery						√		

BMI, body mass index; GBT, gradient boosted trees; LR, linear regression; RMSE, root mean squared error; RF, random forest; SVR, support vector machines regression; VER, voting ensemble for regression.

^a^ Obtained by cross-validation in the training set (*n* = 3,803) during model training (mean ± standard deviation).

^b^ RMSE of the final model when making predictions on the test set (*n* = 951).

## Discussion

### Summary of study findings

This prospective cohort study predicted birth weight by using 8 interpretable machine learning models. A total of 24 predictors including socio-demographic, lifestyle, and biomarker features were used to fit the models. Prediction accuracy was better in voting ensemble for regression, linear regression, and SVR. Across all models, gestational age, fetal sex, preterm birth, mother's height, and pre-pregnancy BMI were the 5 most important predictors for infant birth weight. After adding ultrasound-measured indicators of fetal growth into predictors, mother's height, and pre-pregnancy BMI remained important in predicting the outcome.

### Comparison with other studies

Accurate prediction of birth weight is challenging. On one hand, many factors play a role in determining birth weight. Factors included, but are not limited to, genetic, environmental, and gestational factors. On the other hand, complex relationship beyond the linear relationship between the predictors and outcome might exist. Based on a very recent systematic review, only 35.2% studies applied a machine learning model beyond linear regression in the prediction of pregnancy outcomes (6).

To our knowledge, only 3 very recent studies have applied machine learning algorithms in the prediction of birth weight or related outcomes (e.g., low birth weight (12), macrosomia (13), small and large for gestational age (13, 14)). However, 2 studies (12, 13) of them had modest sample size (*n* = 175 and 1,115 respectively), and more importantly, the studies did not interpret results from machine learning models by calculating feature importance or plotting partial dependence plots. Another study (14), in line with ours, has found that delivery history of infant size and pre-pregnancy BMI were among the important predictors for birth weight related outcomes, but this study (14) only compared feature importance qualitatively.

The three important predictors (mother's height, pre-pregnancy BMI, and history of macrosomia delivery) identified in this study have been validated in other studies in the independent study samples, thereby supporting the validity of the models. First, a cross-sectional study involving 1,511 children (<10 years) in Brazil found that low maternal stature was associated with low birth weight (15). Second, higher mother's pre-pregnancy BMI has been identified as a strong predictor for large for gestational age at birth, based on a meta-analysis of 265,270 individual participant data from 39 cohorts (16). The mechanisms underlying the association of maternal BMI and infant birth weight may include shared genes between mother and fetuses (17), and an increased placental transfer of nutrients (e.g., glucose, lipids). Third, history of macrosomia delivery has been found to be associated with macrosomia or large for gestational age in a cross-sectional study (18) and a retrospective cohort study (14).

### Strengths and limitations

The study had several strengths. First, this is a prospective cohort study, making the association between predictors and birth weight less likely to be inversely causal. Second, this study included many predictors covering not only maternal demographic features, but also father's BMI, and important biomarkers such as hemoglobin, lipids, and glucose, et al. Third, the study used feature importance algorithms and partial dependence plots, increasing the interpretability of machine learning models.

Nevertheless, the study also had some limitations. Sample size of this study was not large enough to accurately fit more parameters in machine learning models as compared to conventional regression models. This might at least partly interpret the unsatisfactory accuracy of the model measured by RMSE (≈370), which means the mean difference between predicted and true values is 370 gram (accounts for 11% of the mean birth weight). Additionally, findings from this pilot study should be interpreted cautiously, and the results will be tested in an external independent dataset in the future.

### Research implications

Considering complex relationship inherent in pregnancy predictors, researchers have called for using both linear regression and other machine learning algorithms in the prediction of pregnancy outcomes. Our study stands for a first step towards the application of machine learning algorithms into prediction of birth weight. Findings of our study indicate that machine learning algorithms are feasible in the present prediction task. Using more features and trying more combinations of hyperparameters in a larger sample can establish a more accurate prediction model in the future. This method may have important practical significance in predicting fetal growth during pregnancy.

## Conclusion

Mother's height and pre-pregnancy BMI were identified as important predictors for infant birth weight. Interpretable machine learning is a promising tool in the prediction of birth weight.

## Data Availability

The original contributions presented in the study are publicly available. This data can be found here: https://figshare.com/articles/software/Python_code_for_Interpretable_machine_learning_to_identify_important_predictors_of_birth_weight_py/21445404.
