# Supplementary material for: Interpretable machine learning to identify important predictors of birth weight: A prospective cohort study
Source: Front Pediatr. 2022 Nov 11;10:899954. doi: 10.3389/fped.2022.899954 (PMC9691849; doi:10.3389/fped.2022.899954)
Supplement: Supplementary file 1 [file Datasheet1.pdf]

Supplemental material

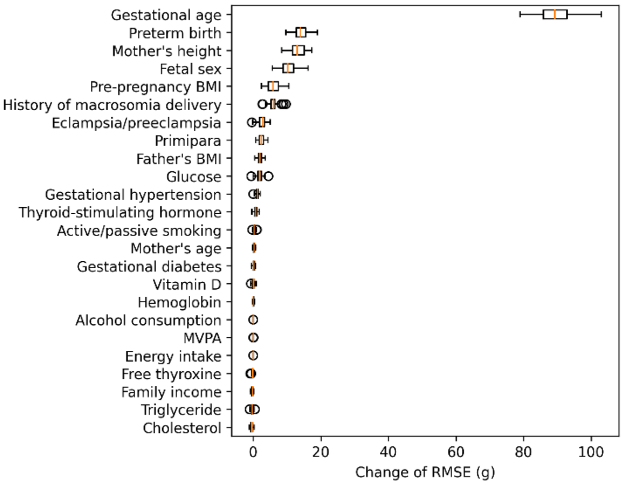

(A)

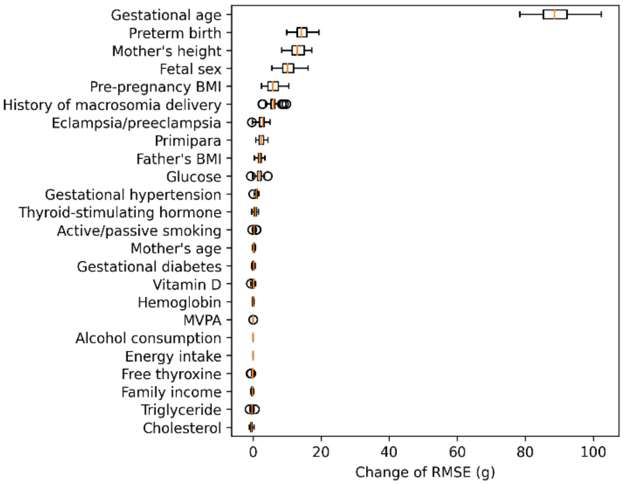

(B)

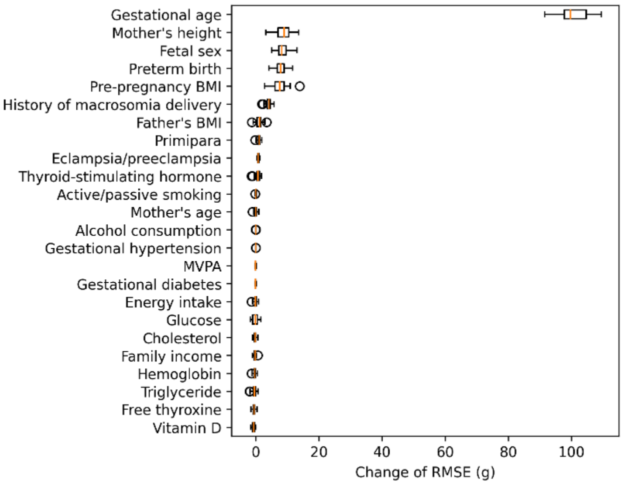

(C)

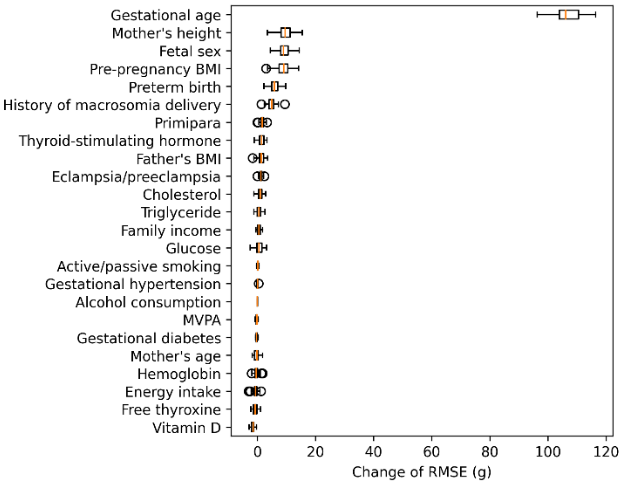

(D)

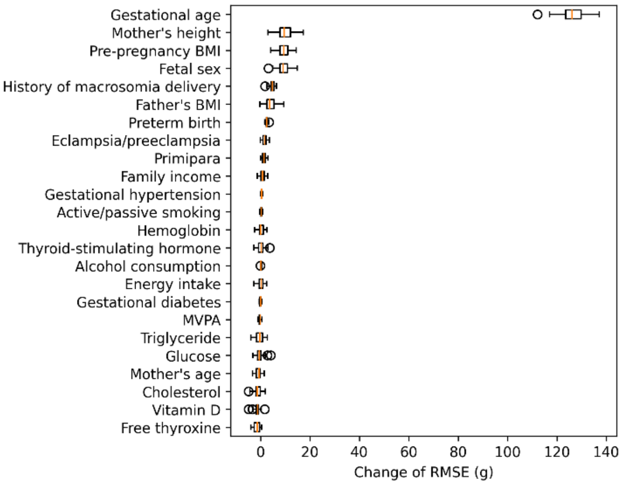

(E)

Supplemental Figure 1 Permutation importance of predictors in the ridge regression (A), lasso regression (B), random forest (C), AdaBoost (D), and gradient boosted tree (E) models (Calculation of permutation importance score was repeated for 50 times, and the box-plot for each predictor showed the distribution of 50 permutation scores)

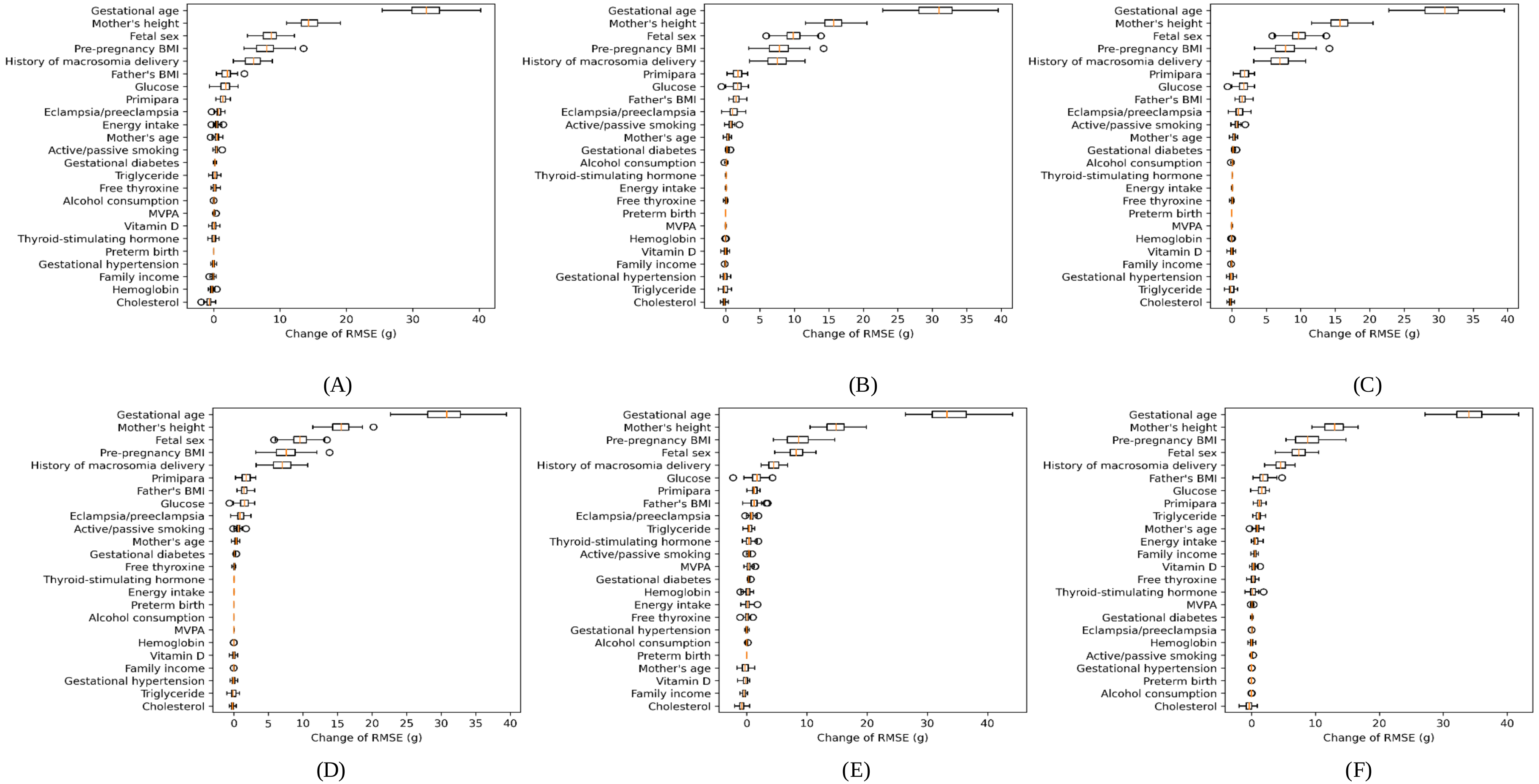
Gestational age
Mother's height
Fetal sex
Pre-pregnancy BMI
History of macrosomia delivery
Father's BMI
Glucose
Primipara
Eclampsia/preeclampsia
Energy intake
Mother's age
Active/passive smoking
Gestational diabetes
Triglyceride
Free thyroxine
Alcohol consumption
MVPA
Vitamin D
Thyroid-stimulating hormone
Preterm birth
Gestational hypertension
Family income
Hemoglobin
Cholesterol
0 10 20 30 40
Change of RMSE (g)
(A)
Gestational age
Mother's height
Fetal sex
Pre-pregnancy BMI
History of macrosomia delivery
Primipara
Glucose
Father's BMI
Eclampsia/preeclampsia
Active/passive smoking
Mother's age
Gestational diabetes
Alcohol consumption
Thyroid-stimulating hormone
Energy intake
Free thyroxine
Preterm birth
MVPA
Hemoglobin
Vitamin D
Family income
Gestational hypertension
Triglyceride
Cholesterol
0 5 10 15 20 25 30 35 40
Change of RMSE (g)
(B)
Gestational age
Mother's height
Fetal sex
Pre-pregnancy BMI
History of macrosomia delivery
Primipara
Glucose
Father's BMI
Eclampsia/preeclampsia
Active/passive smoking
Mother's age
Gestational diabetes
Alcohol consumption
Thyroid-stimulating hormone
Energy intake
Free thyroxine
Preterm birth
MVPA
Hemoglobin
Vitamin D
Family income
Gestational hypertension
Triglyceride
Cholesterol
0 5 10 15 20 25 30 35 40
Change of RMSE (g)
(C)
Gestational age
Mother's height
Fetal sex
Pre-pregnancy BMI
History of macrosomia delivery
Primipara
Father's BMI
Glucose
Eclampsia/preeclampsia
Active/passive smoking
Mother's age
Gestational diabetes
Free thyroxine
Thyroid-stimulating hormone
Energy intake
Preterm birth
Alcohol consumption
MVPA
Hemoglobin
Vitamin D
Family income
Gestational hypertension
Triglyceride
Cholesterol
0 5 10 15 20 25 30 35 40
Change of RMSE (g)
(D)
Gestational age
Mother's height
Pre-pregnancy BMI
Fetal sex
History of macrosomia delivery
Glucose
Primipara
Father's BMI
Eclampsia/preeclampsia
Triglyceride
Thyroid-stimulating hormone
Active/passive smoking
MVPA
Gestational diabetes
Hemoglobin
Energy intake
Free thyroxine
Gestational hypertension
Alcohol consumption
Preterm birth
Mother's age
Vitamin D
Family income
Cholesterol
0 10 20 30 40
Change of RMSE (g)
(E)
Gestational age
Mother's height
Pre-pregnancy BMI
Fetal sex
History of macrosomia delivery
Father's BMI
Glucose
Primipara
Triglyceride
Mother's age
Energy intake
Family income
Vitamin D
Free thyroxine
Thyroid-stimulating hormone
MVPA
Gestational diabetes
Eclampsia/preeclampsia
Hemoglobin
Active/passive smoking
Gestational hypertension
Preterm birth
Alcohol consumption
Cholesterol
0 10 20 30 40
Change of RMSE (g)
(F)

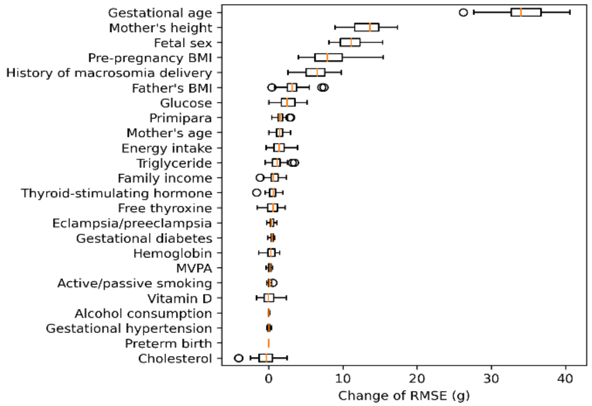

(G)

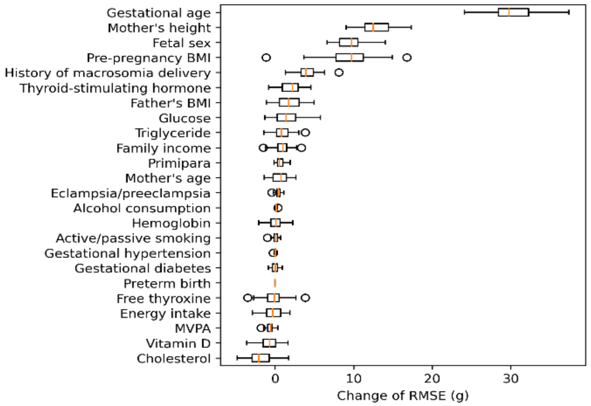

(H)

Supplemental Figure 2 Permutation importance of predictors in the voting ensemble for regression (A), linear regression (B), ridge regression (C), lasso regression (D), support vector machines (E), random forest (F), AdaBoost (G), AND gradient boosted tree (H) models when excluding preterm-birth and/or small-for-gestational-age infants

(Calculation of permutation importance score was repeated for 50 times, and the box-plot for each predictor showed the distribution of 50 permutation scores)

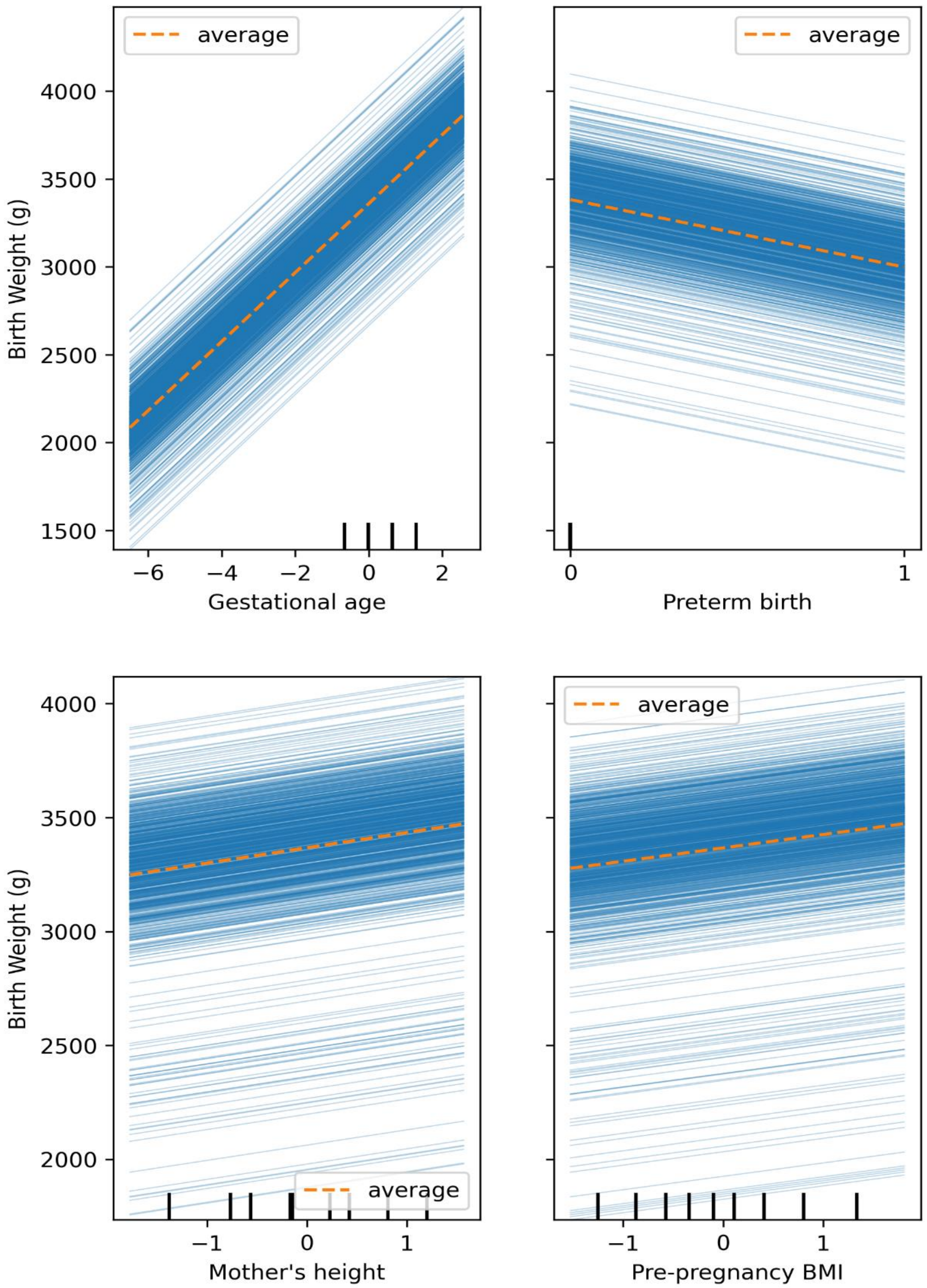

Supplemental Figure 3 Partial dependence plots of gestational age, preterm birth, mother's height, and pre-pregnancy BMI in the prediction of birth weight in the linear regression model (gestational age, mother's height, and pre-pregnancy BMI were standardized)

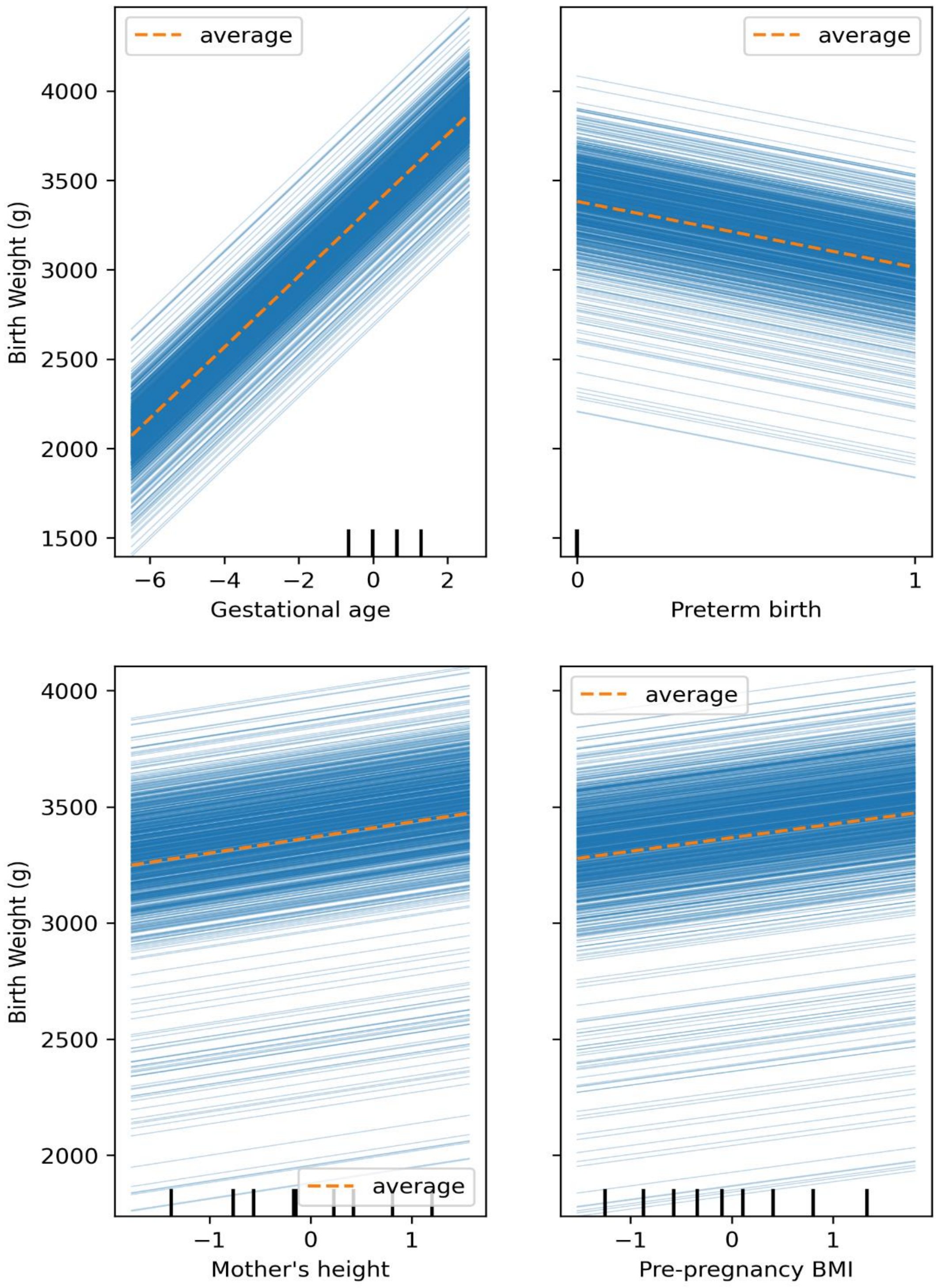

Supplemental Figure 4 Partial dependence plots of gestational age, preterm birth, mother’s height, and pre-pregnancy BMI in the prediction of birth weight in the ridge regression model (gestational age, mother’s height, and pre-pregnancy BMI were standardized)

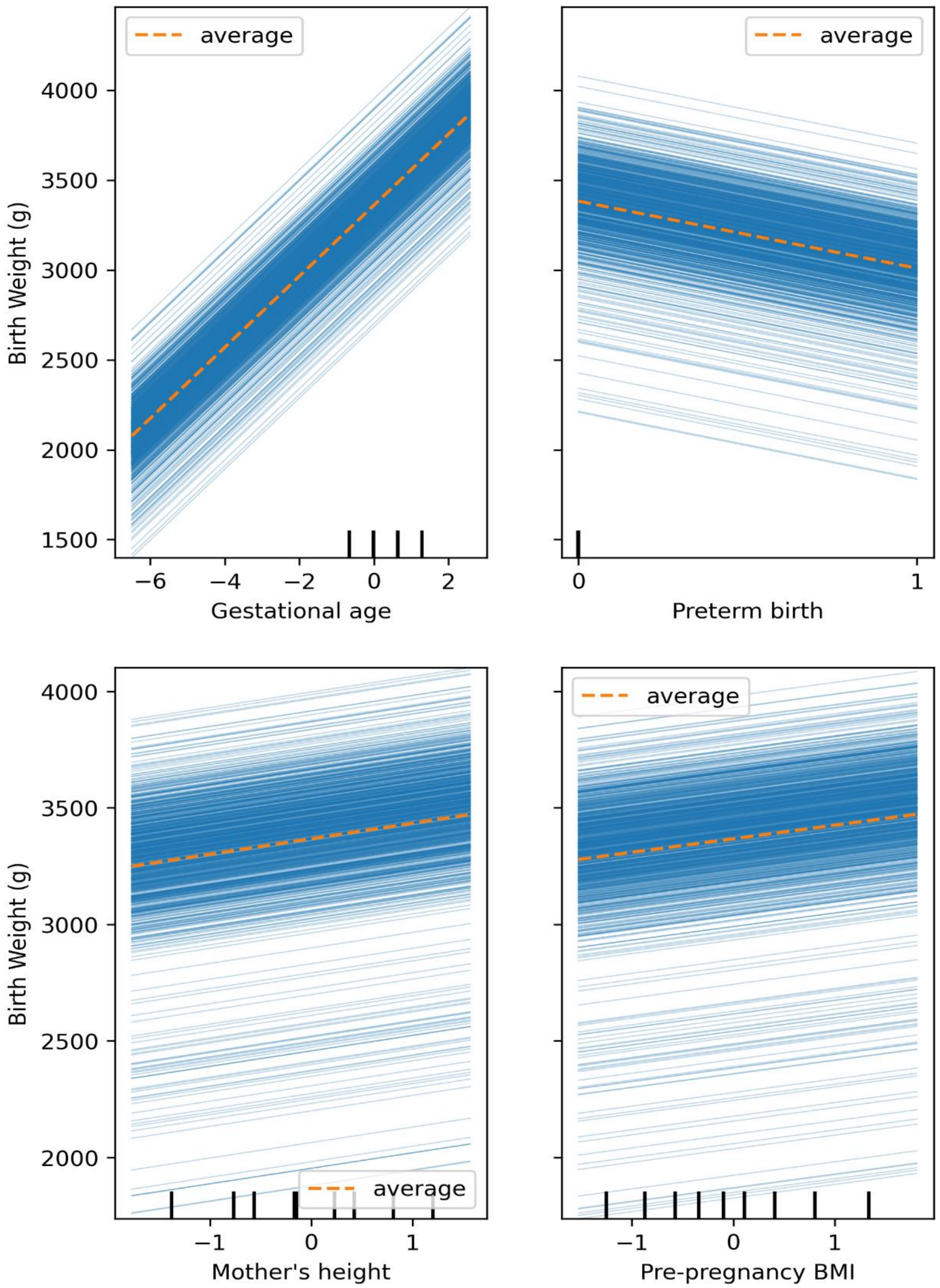

Supplemental Figure 5 Partial dependence plots of gestational age, preterm birth, mother’s height, and pre-pregnancy BMI in the prediction of birth weight in the lasso regression model (gestational age, mother’s height, and pre-pregnancy BMI were standardized)

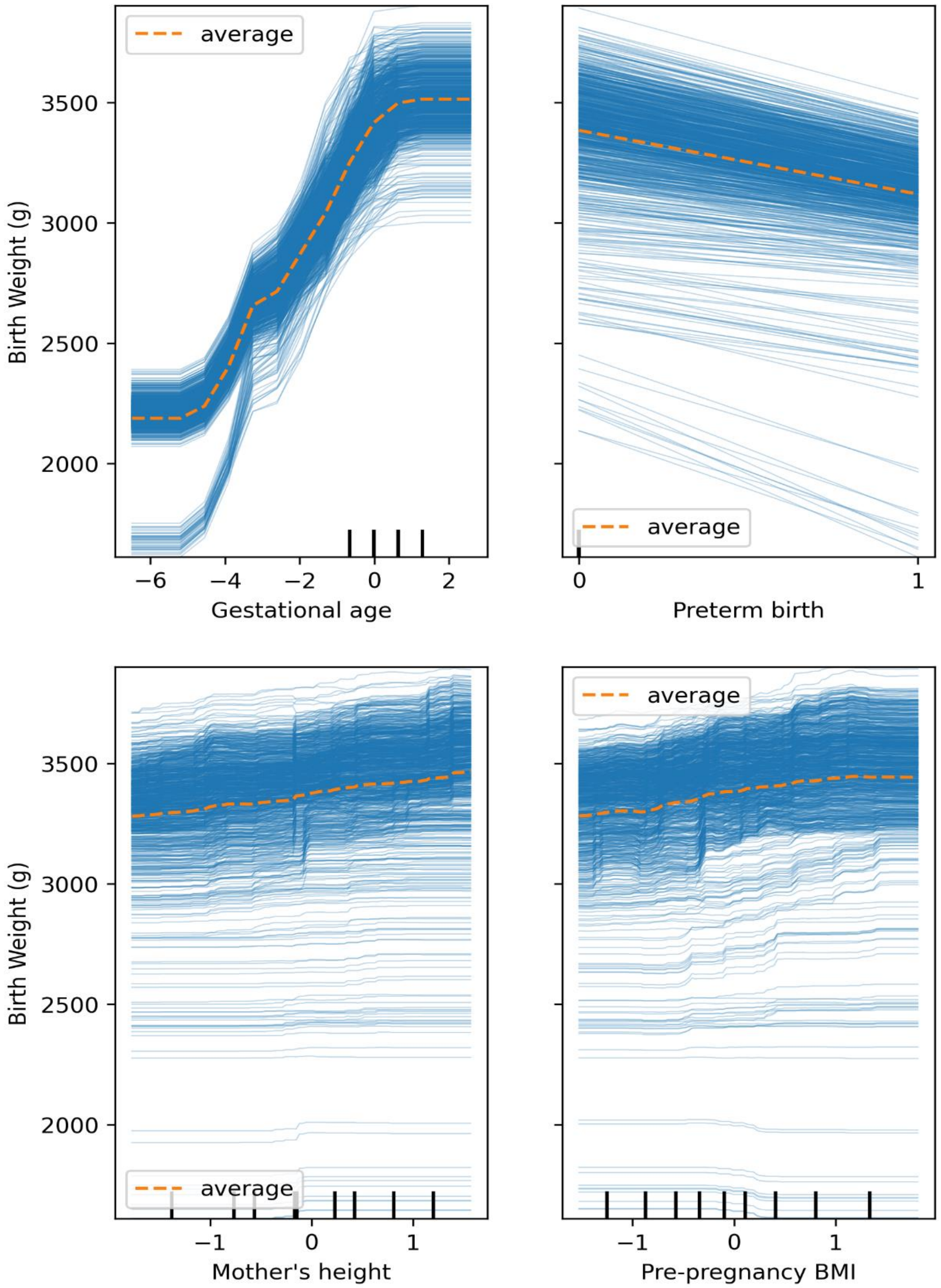

Supplemental Figure 6 Partial dependence plots of gestational age, preterm birth, mother’s height, and pre-pregnancy BMI in the prediction of birth weight in the random forest regression model (gestational age, mother’s height, and pre-pregnancy BMI were standardized)

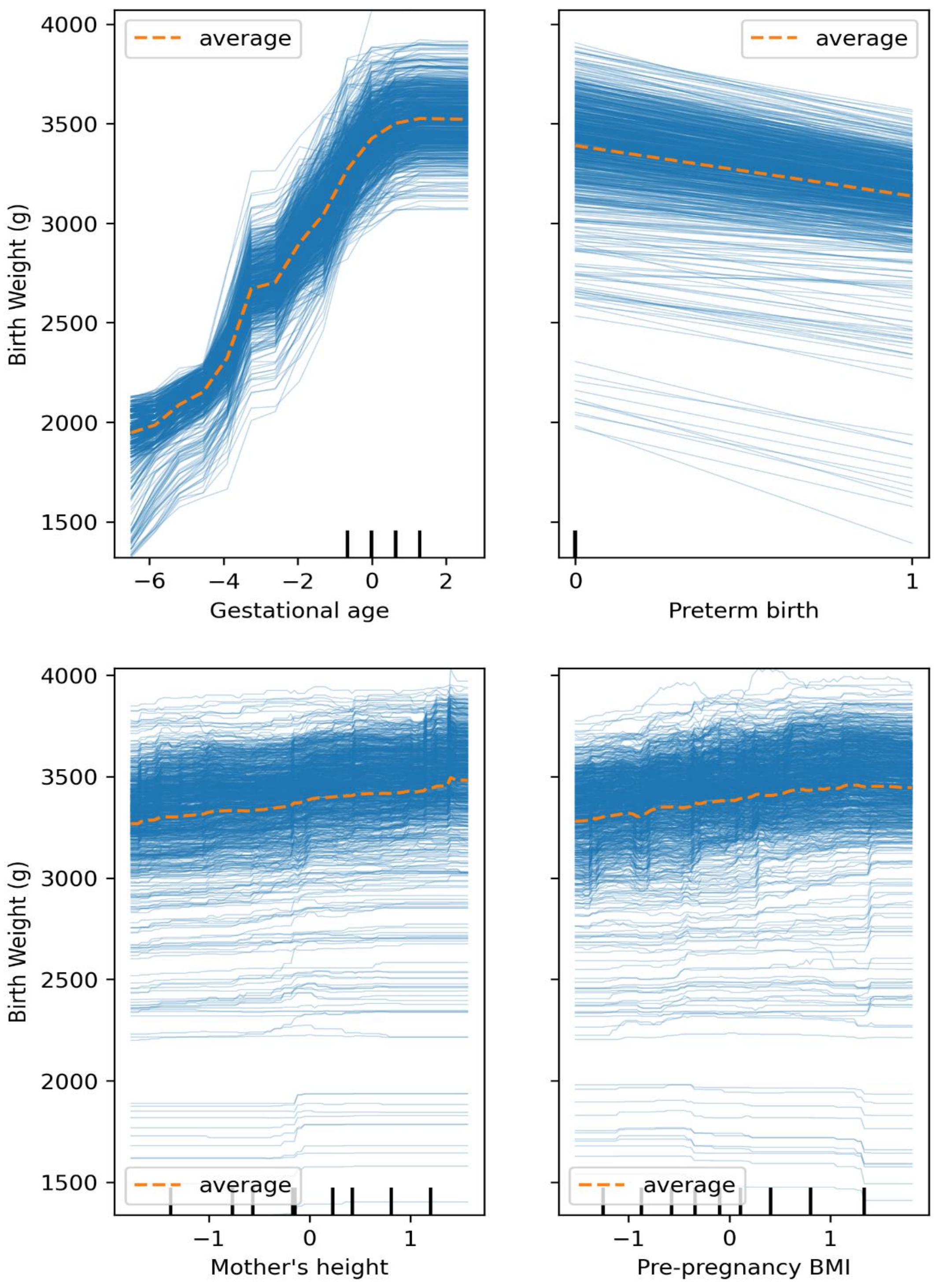

Supplemental Figure 7 Partial dependence plots of gestational age, preterm birth, mother’s height, and pre-pregnancy BMI in the prediction of birth weight in the AdaBoost model (gestational age, mother’s height, and pre-pregnancy BMI were standardized)

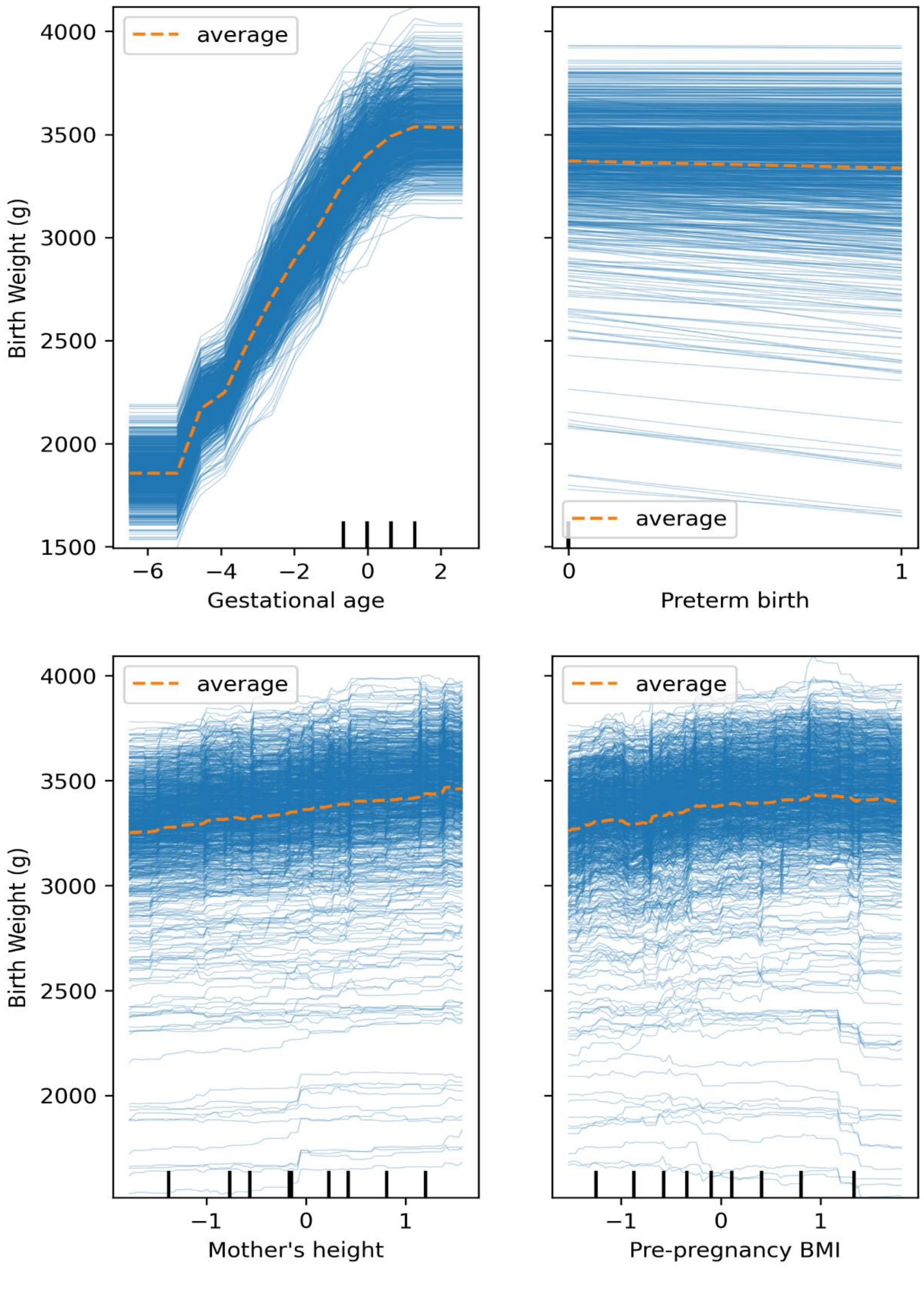

Supplemental Figure 8 Partial dependence plots of gestational age, preterm birth, mother’s height, and pre-pregnancy BMI in the prediction of birth weight in the gradient boosted trees model (gestational age, mother’s height, and pre-pregnancy BMI were standardized)
